# Structural connectivity of cytoarchitectonically distinct human left temporal pole subregions: a diffusion MRI tractography study

**DOI:** 10.3389/fnana.2023.1240545

**Published:** 2023-11-28

**Authors:** Takeshi Sasaki, Nikos Makris, Martha E. Shenton, Peter Savadjiev, Yogesh Rathi, Ryan Eckbo, Sylvain Bouix, Edward Yeterian, Bradford C. Dickerson, Marek Kubicki

**Affiliations:** ^1^Psychiatry Neuroimaging Laboratory, Brigham and Women’s Hospital, Harvard Medical School, Boston, MA, United States; ^2^Center for Morphometric Analysis, Department of Psychiatry, Neurology, and Radiology Services, Massachusetts General Hospital, Harvard Medical School, Charlestown, MA, United States; ^3^Department of Psychiatry and Behavioral Sciences, Tokyo Medical and Dental University Graduate School, Tokyo, Japan; ^4^Department of Radiology, Brigham and Women’s Hospital, Harvard Medical School, Boston, MA, United States; ^5^Frontotemporal Disorders Unit, Massachusetts General Hospital, Harvard Medical School, Charlestown, MA, United States; ^6^Department of Software Engineering and Information Technology, École de Technologie Supérieure, Montréal, QC, Canada; ^7^Department of Psychology, Colby College, Waterville, ME, United States

**Keywords:** temporal pole, structural connectivity, diffusion tensor imaging, tractography, cytoarchitecture, default mode network, semantic dementia

## Abstract

The temporal pole (TP) is considered one of the major paralimbic cortical regions, and is involved in a variety of functions such as sensory perception, emotion, semantic processing, and social cognition. Based on differences in cytoarchitecture, the TP can be further subdivided into smaller regions (dorsal, ventrolateral and ventromedial), each forming key nodes of distinct functional networks. However, the brain structural connectivity profile of TP subregions is not fully clarified. Using diffusion MRI data in a set of 31 healthy subjects, we aimed to elucidate the comprehensive structural connectivity of three cytoarchitectonically distinct TP subregions. Diffusion tensor imaging (DTI) analysis suggested that major association fiber pathways such as the inferior longitudinal, middle longitudinal, arcuate, and uncinate fasciculi provide structural connectivity to the TP. Further analysis suggested partially overlapping yet still distinct structural connectivity patterns across the TP subregions. Specifically, the dorsal subregion is strongly connected with wide areas in the parietal lobe, the ventrolateral subregion with areas including constituents of the default-semantic network, and the ventromedial subregion with limbic and paralimbic areas. Our results suggest the involvement of the TP in a set of extensive but distinct networks of cortical regions, consistent with its functional roles.

## Introduction

The temporal pole (TP), the rostralmost region of the temporal lobe anterior to the limen insulae (Ding et al., 2009), is one of the major paralimbic cortical regions (Mesulam, 2000) involved in a variety of functions, including sensory perception, emotion, semantic processing, autobiographical memory, and social cognition (Olson et al., 2013; Ralph et al., 2017; Gonzalez Alam et al., 2021; Herlin et al., 2021; Setton et al., 2022). The TP has been implicated in a number of diseases such as semantic dementia (Acosta-Cabronero et al., 2011; Mesulam et al., 2013; Collins et al., 2017), Alzheimer’s disease (Desikan et al., 2008; La Joie et al., 2014), schizophrenia (Kasai et al., 2003), rapid cycling bipolar disorder (Murai and Fujimoto, 2003), and attention-deficit hyperactive disorder (Fernandez-Jaen et al., 2014). Even though it was originally described as a uniform area by Brodmann (area 38), the multimodal functional nature of the TP suggests its heterogeneity and existence of subdivisions with different connectivity profiles. Elucidating subdivisions of the TP along with their individual connectivity profiles might help in understanding the role the TP plays in information processing and integration.

A recent histological study by Ding et al. (2009) has revealed that the TP comprises at least seven subregions of different cytoarchitectonic signature. The study demonstrated the heterogeneity of the TP where agranular and dysgranular cells appear medially, and granular cells appear laterally and dorsally. A gradient towards an increasing isocortical appearance from medial to lateral TP has also been reported by Blaizot et al. (2010). In parallel to our increasing understanding of TP cytoarchitecture, neuroimaging studies have begun to reveal *in vivo* TP connectivity. Pascual and colleagues used resting-state functional magnetic resonance imaging to show that the human TP can be divided into four subregions with distinct resting-state functional connectivity profiles (Pascual et al., 2013). Furthermore, Binney and colleagues used diffusion tensor imaging (DTI) to show structural connectivity between the temporal pole and a portion of the inferior frontal lobe (Binney et al., 2012). Other researchers utilized DTI and probabilistic tractography, suggesting subregions of the TP with distinct structural connectivity profiles (Fan et al., 2013; Papinutto et al., 2016).

Although the aforementioned studies have explored to a certain extent the structural connectivity between the TP and a set of predetermined target regions (Binney et al., 2012; Fan et al., 2013; Papinutto et al., 2016), the comprehensive structural connectivity pattern of the TP has yet to be determined with clarity. Moreover, the relationship between TP structural and functional connectivity maps remains to be addressed. Elucidating the comprehensive structural connectivity of the TP subregions would be an important step for understanding to what degree structural and functional connectivities are closely related and how to interpret neuroanatomically the functional heterogeneity of the TP (Damoiseaux and Greicius, 2009; Honey et al., 2009; van den Heuvel et al., 2009). Although recent studies have revealed that multiple functional networks and fiber pathways connect with the TP in a region-specific manner (Jouen et al., 2018), their precise relationships are not clear, in part due to insufficient information about the comprehensive structural connectivity of the TP.

Additionally, the relationship between TP subregional cytoarchitecture and its structural connectivity profile has not been explored completely. Revealing structural connectivity patterns of morphologically defined, and cytoarchitectonically distinct TP subregions could add important information to further our understanding of TP connectivity. To our knowledge, only one study has performed tractography on TP subregions using specific seeds, which were generated following morphological criteria of cerebral anatomy, namely the sulcal and gyral morphology, and without any *a priori* information on the seeds’ connectivity outside the TP. However, this study by Binney et al. (2012) was performed in a relatively small set of subjects and did not address the cytoarchitectonic affiliation of the seeds. Two more recent DTI studies (Fan et al., 2013; Papinutto et al., 2016) aimed to parcellate the TP based on its structural connectivity with areas outside of it; the resultant parcellated TP subregions were used as seeds for tractography that explored the difference in structural connectivity across the seeds in further detail. Thus, in these studies, a difference in structural connectivity patterns across the seeds had been assumed prior to the performance of tractography.

In addition to elucidating the normal functions of the TP, information about the comprehensive structural connectivity of the TP is critical for understanding disease progression that involves the TP such as semantic dementia. In semantic dementia, degenerative processes may spread through fiber pathways from specific subregions of the TP to their interconnected cortical areas (Acosta-Cabronero et al., 2011; Collins et al., 2017). To validate this hypothesis, a map of the comprehensive structural connectivity from individual TP subregions is needed. In this regard, the default-semantic network (Pascual et al., 2013), one of the networks shown to be connected with the TP, and the clinical consequence of its damage is of particular interest; however, structural connectivity between the TP and the constituents of the default-semantic network has not been fully elucidated.

The aim of the current study was to delineate the comprehensive structural connectivity of cytoarchitectonically distinct TP subregions. To this end, we analyzed 3-Tesla DTI scans in 31 subjects. We utilized FreeSurfer cortical parcellation to create three left TP cortical regions of interest (ROIs) with distinct cytoarchitecture and used these ROIs as seeds for structural connectivity analyses of the left TP. The left TP was chosen because of evidence for its prominent involvement in semantic dementia (Binney et al., 2010; Olson et al., 2013). Building upon previous work of our group as reported by Pascual et al. (2013), in this study we hypothesized that, by exploring the comprehensive structural connectivity of the left TP subregions, we can detect their structural connectivity to regions constituting brain networks that are related to known TP functions, such as language, emotion, and semantic cognition.

## Materials and methods

### Participants

Forty-one healthy subjects were recruited using advertisements. The local IRB committees at Brigham and Women’s Hospital and VA Boston approved the study, and written informed consent was obtained from all subjects. All procedures performed in this study were in accordance with the ethical standards of the institutional research committee and with the ethical principles of the Helsinki declaration. All subjects were right-handed and had no history of psychiatric or neurological abnormalities. Of the initial 41 subjects, 10 failed to meet the quality criteria for inclusion of T1-weighted image parcellation and were further excluded (see details below). Thus, 31 subjects comprised the sample for this investigation [mean age = 36.2 ± 11.2 (SD) years old; age range = 20–54 years old; 28 males and 3 females].

### Image acquisition

Structural T1- and T2-weighted images (T1WI and T2WI) and diffusion weighted images (DWI) were acquired using a 3-Tesla whole body MRI Echospeed system (General Electric Medical Systems, Milwaukee, WI) with 8 channel coil and ASSET (Array Spatial Sensitivity Encoding techniques, GE) with a SENSE factor (speed-up) of 2.

The structural data acquisition included two MRI pulse sequences: spoiled gradient-recalled acquisition (fastSPGR) [TR = 7.4 ms, TE = 3 ms, TI = 600, 10 degree flip angle, 25.6 cm^2^ field of view (FOV), matrix = 256 × 256] producing T1W images, and XETA (eXtended Echo Train Acquisition), which produced a series of contiguous T2-weighted images (TR = 2,500 ms, TE = 80 ms, 25.6 cm^2^ FOV). Both structural acquisitions resulted in 1 × 1 × 1 mm^3^ voxels.

Diffusion-weighted imaging (DWI) was performed using an echo planar imaging (EPI) DWI sequence (TR = 17,000 ms, TE = 78 ms, FOV = 24 cm, matrix = 144 × 144, 1.7 mm slice thickness). A double echo option was used to reduce eddy-current related distortions. Acquisitions used 51 gradient directions with *b* = 900 s/mm^2^ and 8 baseline scans with *b* = 0. All scans had 85 axial slices and covered the whole brain. The original GE sequence was modified to increase spatial resolution and to minimize further image artifacts, and resulted in 1.7 × 1.7 × 1.7 mm^3^ voxel size.

### Creation of left TP subregional seeds

The whole brain parcellation on T1WI was performed using FreeSurfer version 5.3^1^ on a Fedora 13 Linux workstation. The parcellation results of the left temporal pole (TP) for all subjects were visually inspected, and 10 subjects were excluded because of the poor quality of the parcellation, especially the rostral portion of the left TP. This unusually high exclusion rate reflects the fact that the TP is one of the areas where parcellation errors occur at a relatively high rate (Desikan et al., 2006).

**Figure 1:**
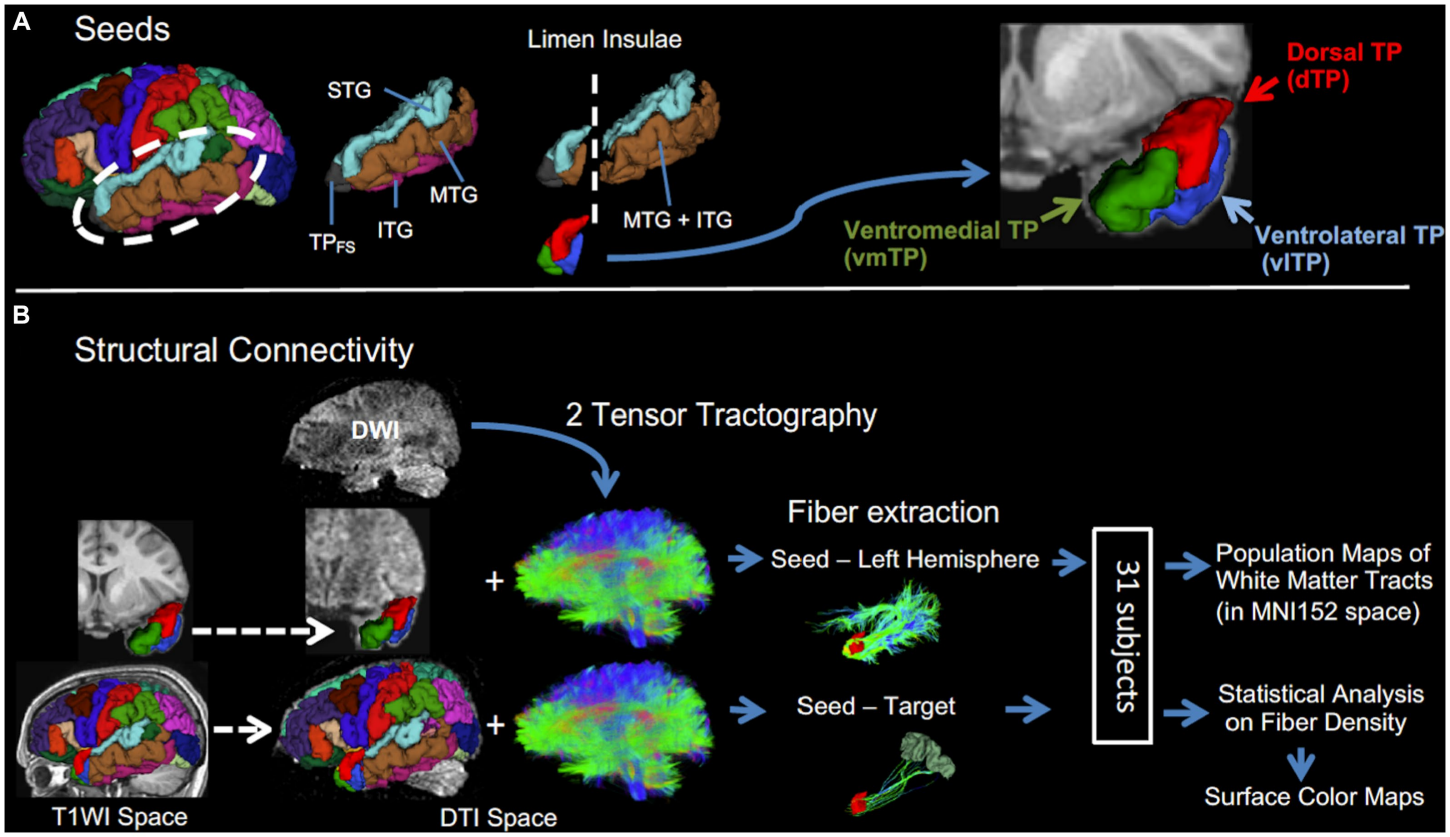
A schematic description of the procedure. **(A)** Left temporal pole subregional seeds (dTP, vlTP, and vmTP) were created in temporal lobe cortices anterior to the limen insulae. **(B)** Structural connectivity analysis. Two-tensor tractography was performed to obtain fibers of the whole brain. Then, from the whole brain tracts, fibers were extracted in two ways: (1) between each seed and the entire left hemisphere and (2) between each seed-target pair. Dashed arrows indicate spatial registration.

For the remaining 31 subjects, the left TP subregional seeds were created for each subject separately utilizing the following four cortical ROIs from FreeSurfer parcellation: the left superior temporal gyrus (STG), left middle temporal gyrus (MTG), left inferior temporal gyrus (ITG) and left “temporal pole.” Of note, the ROI “temporal pole” from FreeSurfer parcellation (TP_FS_) is not equal to the entire TP as investigated in the current study; rather it represents the rostral portion of the TP (Figure 1). Among these four ROIs, only the areas anterior to the limen insulae were used for the creation of TP seeds as follows: first, a mask covering the entire left TP in Montreal Neuroimaging Institute (MNI) 152 space (*y* ≧ 6) was created using 3D Slicer.^2^ Second, the mask was registered to individual T1WI space using a combination of non-linear transformation between MNI to individual T2WI by Advanced Normalization Tools (ANTs) and a linear transformation between individual T2WI and T1WI by FMRIB’s Linear Image Registration Tool (FLIRT) using FMRIB Software Library (FSL) version 4.1.6.^3^ Third, this mask was applied to individual FreeSurfer parcellation, keeping only regions inside the mask, i.e., the area anterior to the limen insulae. Since the cytoarchitecture of the rostral MTG and ITG is identical (Ding et al., 2009), those two labels were merged. The above procedures resulted in three TP subregional seeds for each subject: the dorsal TP (dTP), ventrolateral TP (vlTP), and ventromedial TP (vmTP) seeds, which correspond to the areas of the STG, the MTG merged with ITG, and the TP_FS_, respectively. The dorsal seed (dTP) includes the rostral STG bounded rostrally by the rostral extent of the superior temporal sulcus, medially by the Sylvian fissure, and laterally by the superior temporal sulcus (Desikan et al., 2006). The dTP corresponds to area TAr according to Ding et al. (2009). The ventrolateral seed (vlTP) comprises the rostral MTG merged with the ITG, bounded rostrally by the rostral extent of superior temporal sulcus, medially by the occipitotemporal sulcus, and laterally by the inferior temporal sulcus. The vlTP corresponds to area TE of Ding et al. (2009). The ventromedial TP seed (vmTP) corresponds to the TP_FS_ (the TP from Freesurfer parcellation) and lies in the most anterior portion of the TP. The vmTP’s caudal boundary is the entorhinal cortex that begins with the appearance of the collateral sulcus posteriorly. The vmTP corresponds to area TG of Ding et al. (2009) cytoarchitectonically, the dTP and vlTP are granular, whereas the vmTP is dysgranular (Ding et al., 2009).

### DTI preprocessing

First, the diffusion data were corrected for motion artifacts and eddy current distortions by performing an affine registration of all images to the first b0 image, using FMRIB’s Linear Image Registration Tool (FLIRT). Then, non-brain areas were removed using the automated masking method in 3D Slicer and subsequent manual editing.

### Two-tensor tractography

An unscented Kalman filter (UKF) based multi-tensor tractography algorithm developed in our laboratory was used (Malcolm et al., 2010; Rathi et al., 2011).^2^^,^^4^ It has been shown to be advantageous in delineating crossing fiber regions, with robust estimation of the model parameters (Fillard et al., 2011). This method was used to obtain fibers from the whole brain. Seeding was done in all the voxels where single tensor FA was greater than 0.18. Each voxel was randomly seeded 10 times and each fiber tract was traced from seed to termination, with the termination criterion of FA <0.15 for the primary tensor component (most consistent with the tracking direction). The resulting whole brain tractography was then used to extract specific fiber tracts (see fiber extraction section below).

### Creation of target regions for DTI

Target regions for tractography included all the left cortical ROIs from the FreeSurfer parcellation except for the temporal pole; and two subcortical ROIs including the left amygdala and left hippocampus (35 ROIs in total). Among these ROIs, the following five were modified because they have overlap with the TP subregional seeds: from the STG, MTG, and ITG, areas anterior to the limen insulae were removed because these areas are constituents of TP subregional seeds. In some cases where rostral tips of the entorhinal cortex and fusiform gyrus extended anteriorly beyond the level of the limen insulae, those areas were also removed. Three TP seeds and all target regions in individual T1WI space were then registered to DTI space using non-linear transformation (ANTs).

### Fiber extraction

Fiber extraction was performed in two different ways. First, to visualize the overall structural connectivity between the left TP subregions and the left hemisphere, fibers were extracted between each of the three seeds and the entire left hemisphere across all subjects. Then, the resultant fibers were processed as follows to yield group-average connectivity maps, i.e., population maps. After fiber extraction, the extracted fibers were registered to MNI152 space by applying non-linear transformations between DTI and T1, and between T1 and MNI (both by ANTs), and then binarized. The binarized fibers across all subjects were averaged for each seed separately, yielding population maps of association fibers from each of the three seeds. Voxels in those maps have a value between 0 and 1. Then the maps were thresholded at 0.2 (20%), an optimal value to remove noise (Powell et al., 2006), to show the overall structural connectivity profile of each seed. The Johns Hopkins University WM tractography atlas (Hua et al., 2008) was used to label the fiber pathways except for the middle longitudinal fascicle, which was identified according to previous reports on this association fascicle (Makris et al., 2013a,b).

Second, to statistically investigate the structural connectivity differences across the TP seeds, fiber traces were extracted between each seed-target pair. Only those that had their end points on the cortical surface of the desired target ROIs were retained. For a given target region, structural connection to the TP was deemed absent if the tractography failed to extract fibers between the target region and all three TP seeds in more than three subjects. We excluded such target regions from the statistical analysis. For the remaining target regions, the number of streamlines was calculated for each seed-target pair, and adjusted by dividing it by the added volume (voxel number) of the connecting two regions, yielding streamline density for each connection, following van den Heuvel et al. (2013). We used streamline density for statistical analyses to examine the structural connectivity difference across the TP seeds (see statistical analysis section for details).

### Statistical analysis

For each target region separately, an effect of seed on streamline density was examined using the Friedman test, a non-parametric version of one-way repeated measures analysis of variance (ANOVA), with seed (dTP, vlTP, or vmTP) as the within-subject factor. For the Friedman test, *p* < 0.002 (0.05/25) was considered statistically significant after Bonferroni’s correction for the number of included target regions (see results). For a target region with a significant effect of TP seeds on streamline density, a post-hoc pairwise Wilcoxon signed-rank test between the levels of the factor (seed) was performed to examine which of the three seeds had a significantly higher streamline density than other seeds to the target region. For the post-hoc pairwise Wilcoxon signed-rank test, *p* < 0.017 (0.05/3) was considered statistically significant after Bonferroni’s correction. Higher streamline density was considered to be a proxy for stronger structural connectivity (van den Heuvel et al., 2013). To visualize the results, 3D color maps on a representational subject’s FreeSurfer parcellation map were created for each pairwise comparison separately. The statistical analyses were performed using R version 3.1.1.^5^

## Results

### Overall structural connectivity

**Figure 2:**
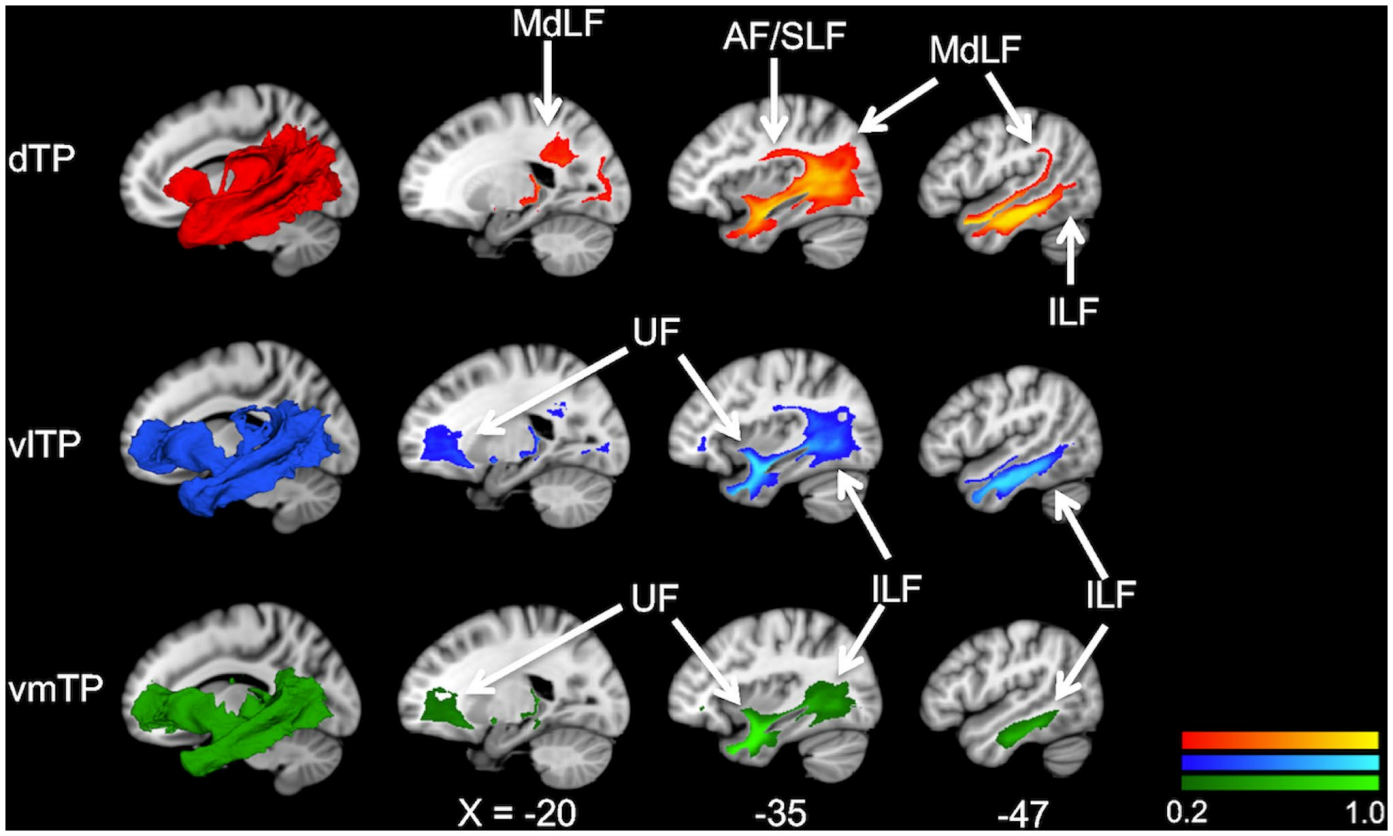
Population maps (group-average connectivity maps) of white matter tracts from the temporal pole subregions, dTP (red), vlTP (blue), and vmTP (green), in the MNI152 template. The color scales show the probability of a voxel belonging to a white matter pathway. The images on the leftmost, superimposed on sagittal slices, are a 3D representation of population maps. AF, arcuate fasciculus; MdLF, middle longitudinal fascicle; UF, uncinate fasciculus; ILF, inferior longitudinal fasciculus.

To examine overall structural connectivity of the TP subregions, population maps (group-average connectivity maps) of white matter bundles for 31 subjects were created for each TP seed separately. The map delineated the trajectories of major association fiber bundles such as the uncinate fasciculus (UF), middle longitudinal fascicle (MdLF), arcuate fasciculus (AF), and inferior longitudinal fasciculus (ILF) (Figure 2). The UF was depicted mainly from the two ventral seeds (vlTP and vmTP), while the MdLF and AF were delineated from the dorsal seed (dTP), indicating a dorsal-ventral segregation. The ILF, however, was observed connecting to all three seeds. The trajectories of these association fibers depicted by the population maps are in agreement with previous reports, indicating that the ILF (Schmahmann et al., 2007; Catani and Thiebaut de Schotten, 2008), UF (Dick and Tremblay, 2012; Von Der Heide et al., 2013; Jung et al., 2017), MdLF (Makris et al., 2013b), and AF (Acosta-Cabronero et al., 2011; Aichelburg et al., 2014; Papinutto et al., 2016) all reach the TP.

### Structural connectivity differences across TP subregions

To explore differences in structural connectivity patterns across the TP subregions, fiber extraction for every seed-target pair, and a subsequent statistical analysis on streamline density was performed. Of the initial 35 target ROIs, 10 were determined not to be connected to the TP based on the criteria mentioned in the method section, and thus were excluded from further analysis. Twenty-five target ROIs were determined as structurally connected to the TP (Table 1) and included in statistical analyses. As previously reported by other researchers (Honey et al., 2009; Wang et al., 2011; Ruschel et al., 2014), streamline density, which reflects strength of structural connectivity and is calculated from streamline counts adjusted by the volume of connecting two regions, showed large interindividual variability. Thus, the median and interquartile ranges were presented for the summary of streamline density (Table 2).

**Table 1:** Target regions with or without structural connection to the temporal pole.

Target regions	Number of subjects without extracted fibers		
	dTP	vlTP	vmTP
**With structural connection to the TP (25 regions)**			
Superior frontal gyrus	1	1	2
Rostral middle frontal gyrus	1	0	1
Medial orbitofrontal cortex	8	2	2
Lateral orbitofrontal cortex	4	0	0
Pars opercularis	2	4	15
Precentral gyrus	0	1	2
Postcentral gyrus	0	4	3
Superior parietal cortex	0	0	1
Inferior parietal cortex	0	0	0
Precuneus cortex	0	0	2
Supramarginal gyrus	0	1	2
Lateral occipital cortex	0	0	0
Pericalcarine cortex	2	6	7
Lingual gyrus	1	1	2
Banks of STS	0	2	8
Superior temporal gyrus	0	0	4
Middle temporal gyrus	0	0	3
Inferior temporal gyrus	0	0	0
Fusiform gyrus	0	0	0
Entorhinal cortex	8	9	0
Parahippocampal gyrus	2	1	0
Isthmus cingulate cortex	0	0	0
Insula	0	0	0
Hippocampus	0	0	0
Amygdala	0	0	0
**Without structural connection to the TP (10 regions)**			
Frontal pole	14	4	6
Caudal middle frontal gyrus	19	17	22
Pars orbitalis	7	3	5
Pars triangularis	8	4	5
Paracentral lobule	12	13	14
Cuneus cortex	6	4	13
Transverse temporal cortex	14	26	27
Rostral anterior cingulate cortex	15	9	14
Caudal anterior cingulate cortex	22	21	22
Posterior cingulate cortex	4	7	10

Each cell shows the number of subjects without extracted fibers between each seed-target pair. If the number is more than three for three TP subregions, then the target region was deemed as without structural connection to the TP. STS, superior temporal sulcus, *N* = 31.

**Table 2:** Structural connectivity differences across the temporal pole subregions.

Target regions		Adjusted streamline count (×10^−3^ number of fibers per voxel)			Friedman test^a^	Post-hoc pairwise Wilcoxon test^b^		
	Abbrev.	dTP	vlTP	vmTP	*χ* ^2^	dTP vs. vlTP	vlTP vs. vmTP	vmTP vs. dTP
Superior frontal gyrus	SFG	5 (7)	14 (15)	8 (15)	22.9^††^	vl^**^	vl^**^	vm^*^
Rostral middle frontal gyrus	RMF	5 (14)	19 (28)	14 (26)	20.9^††^	vl^**^		vm^*^
Medial orbitofrontal cortex	mOFC	4 (4)	15 (24)	10 (9)	36.8^††^	vl^**^		vm^**^
Lateral orbitofrontal cortex	lOFC	6 (11)	32 (68)	23 (47)	33.0^††^	vl^**^		vm^**^
Pars opercularis	PO	5 (12)	3 (4)	3 (4)	14.3^†^		vl^*^	d^*^
Precentral gyrus	PrCG	6 (7)	3 (3)	2 (3)	26.8^††^	d^*^	vl^*^	d^**^
Postcentral gyrus	PoCG	5 (6)	3 (3)	2 (3)	13.5^†^			d^*^
Superior parietal cortex	SPC	25 (26)	12 (12)	7 (9)	35.9^††^	d^**^	vl^**^	d^**^
Inferior parietal cortex	IPC	54 (66)	24 (26)	13 (16)	37.4^††^	d^**^	vl^**^	d^**^
Precuneus cortex	PCUN	29 (24)	14 (13)	6 (5)	43.6^††^	d^**^	vl^**^	d^**^
Supramarginal gyrus	SMG	19 (35)	12 (14)	4 (8)	34.3^††^	d^**^	vl^**^	d^**^
Lateral occipital cortex	LOC	63 (41)	36 (31)	24 (36)	21.7^††^	d^**^		d^**^
Pericalcarine cortex	PCAL	7 (8)	9 (13)	5 (6)	11.9			
Lingual gyrus	LING	12 (21)	13 (23)	7 (9)	14.6^†^		vl^**^	d^*^
Banks of STS	STS	42 (59)	25 (39)	6 (6)	48.0^††^	d^**^	vl^**^	d^**^
Superior temporal gyrus	STG	463 (242)	58 (63)	23 (39)	56.6^††^	d^**^	vl^**^	d^**^
Middle temporal gyrus	MTG	118 (166)	375 (290)	24 (55)	58.1^††^	vl^**^	vl^**^	d^**^
Inferior temporal gyrus	ITG	218 (220)	408 (392)	136 (206)	31.5^††^	vl^**^	vl^**^	
Fusiform gyrus	FFG	135 (171)	209 (300)	310 (228)	15.9^†^	vl^*^		vm^**^
Entorhinal cortex	EC	18 (39)	40 (65)	286 (422)	47.4^††^		vm^**^	vm^**^
Parahippocampal gyrus	PHG	43 (102)	65 (119)	120 (170)	21.7^††^		vm^*^	vm^**^
Isthmus cingulate cortex	ICC	58 (54)	42 (45)	18 (21)	26.0^††^		vl^**^	d^**^
Insula	INS	135 (90)	117 (98)	123 (119)	3.2			
Hippocampus	HPC	315 (151)	483 (354)	438 (405)	14.4^†^	vl^**^		vm^*^
Amygdala	AMY	32 (77)	197 (285)	505 (430)	50.0^††^	vl^**^	vm^**^	vm^**^

For adjusted streamline count, values are median (interquartile range).

a For Friedman test, *p* < 0.002 (0.05/25) was considered statistically significant (^†^*p* < 0.002 and ^††^*p* < 0.0001).

b For Wilcoxon test, *p* < 0.017 (0.05/3) was considered statistically significant (^*^p < 0.017 and ^**^p < 0.001); each cell shows the temporal pole subregion with stronger structural connectivity: d, dorsal TP; vl, ventrolateral TP; vm, ventromedial TP.

**Figure 3:**
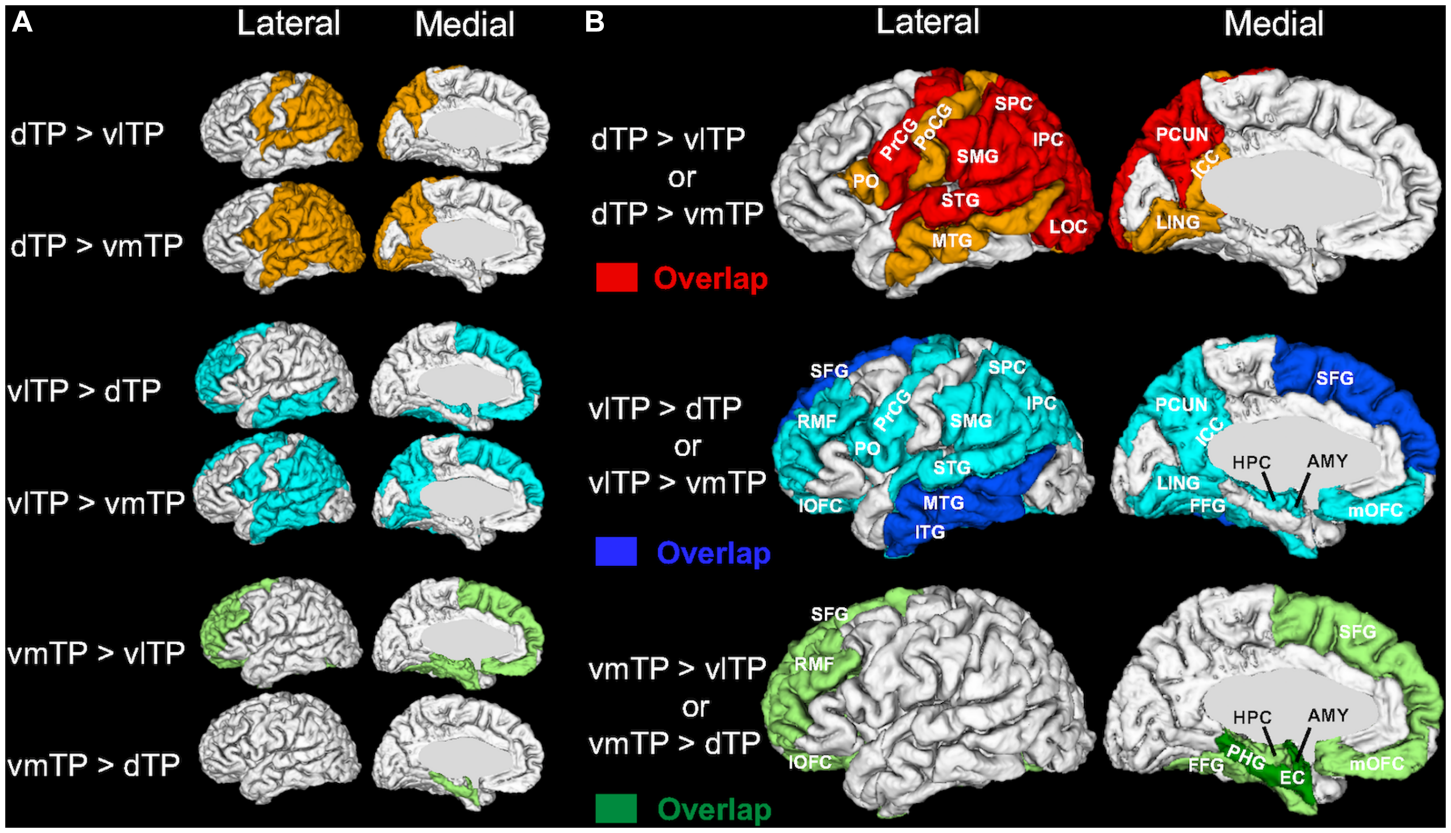
3D surface color maps showing structural connectivity differences across the temporal pole subregions on a representative subject’s brain. **(A)** Target regions with stronger structural connectivity determined by each post-hoc pairwise comparison are shown using light colors. **(B)** The target regions shown in **(A)** are superimposed for each TP seed separately, and overlapping regions are shown in dense colors (dTP, red; vlTP, blue; vmTP, green). Abbreviations for the target regions are shown in Table 2.

A Friedman test (non-parametric one-way repeated measures ANOVA) on the streamline density revealed that there was a significant effect of the TP seeds (dTP, vlTP, or vmTP) on streamline density in all target regions [*χ*^2^ (2) ≧ 13.5, *p* < 0.002] except for the insula and the pericalcarine cortex (Table 2), indicating that structural connectivity patterns are different across the TP seeds. For each target region separately, a subsequent pairwise Wilcoxon signed-rank test examined differences in streamline density across the three TP seeds. The test revealed that for all target regions tested, there was a significant difference in streamline density across the seeds, indicating that structural connectivity differed across the seeds to a wide range of cortical and subcortical areas outside of the TP (Table 2 and Figure 3B). The dTP had stronger structural connectivity with wide areas in the parietal lobe as well as its adjacent areas than the other two seeds. The vlTP showed stronger structural connectivity with wide areas in four cortical lobes, especially with the superior frontal gyrus and lateral temporal areas. The vmTP was revealed to have stronger structural connectivity with medial frontal and temporal areas, with especially strong structural connectivity with the entorhinal cortex, parahippocampal gyrus, and the amygdala.

## Discussion

The aim of the present study was to elucidate the patterns of structural connectivity of the human left temporal pole (TP) subregions with distinct cytoarchitecture. To this aim, based on a recent histological study of the temporal pole (Ding et al., 2009) and utilizing an automatic cortical parcellation technique, we created three cytoarchitectonically distinct cortical TP subregions and subsequently used these as seeds for diffusion tractography. We then recorded and compared tractography profiles between each of the TP subregions, and the entire brain. Our results indicate that each TP subregion has an overlapping yet distinct pattern of extensive structural connections with cortical and subcortical brain areas. We demonstrated these connections in two ways. First, white matter population maps (group-average connectivity maps) (Figure 2) showed four association fiber bundles—the uncinate fasciculus (UF), inferior longitudinal fasciculus (ILF), middle longitudinal fasciculus (MdLF), and arcuate fasciculus (AF)—emanating from the TP with a dorsal-ventral segregation; the dorsal TP has predominant connectivity with the MdLF and AF, whereas the ventral TP has predominant connectivity with the UF. Second, statistical analysis of streamline density comparing structural connectivity strength across the TP subregions revealed differences across the three subregions. Specifically, the dTP showed structural connectivity with the majority of the parietal cortex, the vlTP with widespread frontal, parietal, temporal and occipital cortical areas, and the vmTP with medial frontal and temporal areas (Figure 3). Given the distinct cytoarchitecture of the TP subregions, our results suggest a close relationship between TP subregional cytoarchitecture and structural connectivity with areas outside of the TP. Our results also suggest that the TP has more extensive structural connectivity than has been reported previously, and that this structural connectivity is the basis for the TP’s involvement in a wide range of functions including sensory perception, language, and emotion.

The unique feature of this study compared with previous TP structural connectivity studies is that we used seeds for tractography based solely on the sulco-gyral patterns of the TP, which correspond with the cytoarchitecture of the TP (Ding et al., 2009). We successfully showed that the TP subregions created without prior knowledge of their connectivity profile have distinct structural connectivity patterns. Furthermore, we explored comprehensive structural connectivity of the temporal pole subregions without excluding any cortical target regions from the analysis. Consequently, the number of regions determined to be connected with the left TP was 25 (out of 35 target regions examined), which is very large compared with previous studies in which predetermined target regions were used [i.e., 6 (Binney et al., 2012), 8 (Papinutto et al., 2016), and 17 (Fan et al., 2013) target regions]. The present study builds on these previous TP connectivity studies, and our results for the most part agree with those studies, with a few notable exceptions described below.

### Dorsal temporal pole

The dTP showed stronger structural connectivity with broad areas of the parietal lobe than did the other TP subregions based on the statistical analysis of streamline density (Figure 3). This connectivity seems to be largely through the MdLF, as the population map clearly delineated that MdLF extended from the dTP to the parietal lobe (Figure 2). Our findings are consistent with previous studies that have shown that the MdLF connects the dorsal TP with the parietal lobe (Makris et al., 2013a; Duffau, 2015; Jouen et al., 2018). Makris et al. (2013a) have reported that the MdLF connects the dorsal TP with both the superior and inferior parietal lobes, suggesting that the MdLF plays an important role in language, high order auditory association, and visuospatial and attention functions.

The superior temporal gyrus was also revealed to have strong connection with the dTP. This is consistent with previous findings that the dTP receives input from auditory association areas, implying a critical role in the processing of complex auditory stimuli (Olson et al., 2007).

Strong structural connectivity between the dTP and the lateral occipital lobe was also observed (Figure 3). The population map suggested that this connectivity is most likely via the ILF. Although the ILF is thought to run within the ventral temporal lobe from the occipital lobe to the temporal pole, a previous report using probabilistic tractography suggested a strong connection between the dorsal TP and the occipital lobe via the ILF in humans (Papinutto et al., 2016), which is in line with our results.

It is of note that the AF was delineated from the dorsal TP (Figure 2). Some recent studies indicate that the AF extends anteriorly in the temporal cortex beyond Wernicke’s area and possibly reaches the TP (Saur et al., 2008; Acosta-Cabronero et al., 2011; Papinutto et al., 2016). Whether the AF reaches the TP is a matter of debate (Dick and Tremblay, 2012), because of the possibility of an artifact. Nevertheless, our study suggests that the AF may extend rostrally beyond the traditionally defined language network to reach the TP. Indeed, Papinutto et al. (2016) detected a modest connection between the TP and the inferior frontal gyrus through the AF. In our data, the dTP was connected to pars opercularis in 29 of 31 subjects (Table 1).

### Ventrolateral temporal pole

One of the two ventral TP subregions, the vlTP was shown to have the most widespread structural connectivity profile among the three TP subregions, demonstrating connections to areas in all four cortical lobes (Figure 3). Additionally, it showed stronger structural connectivity with the middle and inferior temporal lobes as well as the superior frontal gyrus than did the other TP subregions (Figure 3). Among the networks that possibly have structural connectivity with the vlTP, the default-semantic network (Pascual et al., 2013) is of particular interest because of the involvement of the vlTP in semantic processing (Binney et al., 2010; Jackson et al., 2016; Mehta et al., 2016). Here we use the term default-semantic network as previously done by Pascual et al. (2013) because the default mode network (Raichle et al., 2001) largely overlaps with the semantic network (Binder et al., 2009; Wei et al., 2012; Humphreys et al., 2015), although the two networks should not be deemed as identical (Jackson et al., 2019).

Indeed, the vlTP showed strong structural connectivity with the constituents of the default-semantic network, including the dorsomedial prefrontal cortex (located within the superior frontal gyrus ROI of the FreeSurfer parcellation), precuneus/posterior cingulate cortex (precuneus and isthmus cingulate cortex ROI), and angular gyrus (inferior parietal cortex ROI) (Figure 3). As illustrated in Figure 3, particularly the connection with the superior frontal gyrus was the strongest for the vlTP among the three TP subregions. The anatomical structure providing the bulk of this connectivity, according to the population maps result, is most likely the uncinate fasciculus (UF) (Figure 2). The UF has been reported to reach the frontal pole (BA 10) (Crosby et al., 1962; Thiebaut de Schotten et al., 2012; Von Der Heide et al., 2013), middle frontal gyrus (Crosby et al., 1962; Hau et al., 2016) and even the superior frontal gyrus (Croxson et al., 2005). It should be noted that the superior frontal gyrus ROI from FreeSurfer used in the current study is large, encompassing such areas as frontal polar and medial prefrontal with which connectivity of the UF has been reported (Crosby et al., 1962; Thiebaut de Schotten et al., 2012; Von Der Heide et al., 2013). The angular gyrus (inferior parietal cortex), one of the other constituents of the default-semantic network, was also shown to have structural connectivity with the vlTP (Figure 3), and this structural connectivity is likely via the ILF (Figure 2). The involvement of the ILF in the connection between the vlTP and the angular gyrus is also suggested by the fact that, in macaque, the ILF has been reported to reach area Opt (Schmahmann et al., 2007; Katsuki and Constantinidis, 2012), a possible macaque homolog of human angular gyrus (Caspers et al., 2011). Even though the anatomical substrate of the structural connectivity between the vlTP and the precuneus/isthmus cingulate cortex was not detected in the present study, overall our results demonstrate structural connectivity between the vlTP and the majority of the cortical areas constituting the default-semantic network.

It should be noted that the structural connectivity from the vlTP is not limited to the cortical areas constituting the default-semantic network. Rather, the vlTP shows structural connectivity with broad areas other than the default-semantic network such as limbic areas, which is also in line with a previous study using functional MRI (Pascual et al., 2013).

### Ventromedial temporal pole

The second ventral subregion of the TP, ventromedial TP (vmTP), was shown to have strong structural connectivity with limbic and paralimibic areas including the amygdala, hippocampus, entorhinal cortex, parahippocampal cortex, and orbitofrontal cortex (Figure 3), which is likely mediated by the UF (Figure 2). The UF gives branches to such areas as the perirhinal cortex and amygdala (Schmahmann and Pandya, 2006) and is involved in emotional processing (Von Der Heide et al., 2013). Our results are in line with previous reports showing the medial TP’s strong structural (Fan et al., 2013) and resting-state functional connectivity (Fan et al., 2013; Pascual et al., 2013) with the limbic-paralimbic areas.

### Cytoarchitecture and connectivity of the TP

The human TP is a complex, transitional cortical region, where many different cortical inputs converge (Ding et al., 2009). Cytoarchitectonically, human TP contains areas corresponding to each of the three cytoarchitectonic cortical types, i.e., agranular, dysgranular, and granular (Ding et al., 2009). Although cytoarchitectonic transitions between subregions separated by sulci and gyri are typically gradual, these TP subregions have broadly different cytoarchitectures (Ding et al., 2009). The dorsal (dTP) and ventrolateral (vlTP) subregions, corresponding to the areas TA and TE, respectively [as described by Ding et al. (2009)], both have granular cytoarchitecture (Ding et al., 2009). By contrast, the ventromedial subregion (vmTP), corresponding to the area TG of Ding et al. (2009), has dysgranular cytoarchitecture (Ding et al., 2009).

Our results demonstrate that the granular subregions of the TP (dorsal and ventrolateral TP) are structurally connected with areas related to phylogenetically more advanced functions such as language, spatial cognition, and semantic processing, whereas the dysgranular TP subregion is structurally connected with phylogenetically less advanced regions such as the limbic network. It should be noted that the TP in the current study included only areas anterior to the limen insulae, excluding the limen insulae itself as well as the areas in fusiform gyrus and entorhinal cortex defined by FreeSurfer parcellation. Consequently, area 36, which is dysgranular (Ding et al., 2009), and area 35, area TI, and the entorhinal cortex, which are agranular (Ding et al., 2009), were not included in the TP in this study, and their structural connectivity was not investigated.

### Connectivity and function of the TP

Our findings are compatible with the view that the TP, based on its structural connections, plays an important role in multiple cognitive functions, including sensory perception, emotion, and semantic processing. More specifically, the TP receives highly processed sensory information (Olson et al., 2007) from visual and auditory areas via ILF and MdLF, respectively, as well as semantic information via connections with the default-semantic network. In semantic processing, the ventrolateral anterior temporal lobes have recently been proposed as cross-modal hubs where all modality-specific information is mediated to form generalized concepts (Ralph et al., 2017). On the other hand, processing of emotional information through limbic networks occurs in the TP, creating emotional tagging (Blaizot et al., 2010) of information received in the TP. Thus, the TP serves as an interface of cognition and emotion (Olson et al., 2007), playing an important role in representing social knowledge (Olson et al., 2007, 2013). Furthermore, through its connection to the prefrontal cortex via the UF, the TP transmits the information to orbitofrontal cortex, playing a critical role in decision-making based on the emotional tone of information (Von Der Heide et al., 2013).

### Limitations

There are several limitations of the current study. The first is the utilization of streamline density as the indirect measure of the strength of structural connectivity. Because streamline density, which is calculated directly from raw streamline count, is inherently susceptible to errors in the fiber orientation due to the presence of noise in the data (Binney et al., 2012; Jones et al., 2013), its use as a measure of the strength of structural connectivity has been a matter of debate (Binney et al., 2012; Jones et al., 2013). Nonetheless, streamline density has been used in previous connectivity studies (Honey et al., 2009; Uddin et al., 2010; van den Heuvel et al., 2013), suggesting its utility as an indirect measure of connection strength. Additionally, our findings are in line with previous studies of the TP structural connectivity using an alternative method [probabilistic tractography (Fan et al., 2013; Papinutto et al., 2016)].

The second limitation is the lack of sophisticated adjustment techniques in our tractography. We have performed the adjustment of the streamline count by the volume of connecting two regions. Since gray matter volume is a product of the surface area and cortical thickness, adjusting for the volume in theory corrects for both. However, since cortical thickness partially drives the strength of structural connectivity (He et al., 2007; Hagmann et al., 2008), ideally, the effect of difference in cortical thickness should not have been excluded. This issue clearly needs more attention, and development of more precise adjustment methods should be addressed in future studies. We did not use state-of -the-art algorithms for improving the accuracy of a streamline reconstruction such as spherical-deconvolution informed filtering of tractograms (Smith et al., 2013). We did not adjust the results of tractography in relation to the distance between a target region and each of the three TP subregions. Although the three subregions are adjacent, there are subtle differences in the distances between each of the three seeds and a given target region. These differences in distance, as well as differences in trajectory between each seed-target pair, might have biased the extraction of streamlines (Jones et al., 2013). However, we did not compare the streamline density across target regions, which would have been much more problematic, given the distance differences.

Third, objection could be raised to our assumption that the cytoarchitecture of the TP subregions is distinct. The TP subregions in this study were defined morphologically for each subject using FreeSurfer segmentation. Because the boundary of TP subregions defined by FreeSurfer match with those of the suggested TP subregional cytoarchitecture (Ding et al., 2009), we believe it is reasonable to assume that the TP subregions defined in this study are cytoarchitectonicaly distinct. However, we acknowledge that our assumption is based on one histological study of the TP (Ding et al., 2009), which also further acknowledges that the transition between subregions is gradual. Utilizing data set such as BigBrain (Amunts et al., 2013), a high-resolution, three-dimensional histological reconstruction of an individual human brain, might be useful to verify the validity of the parcellation of the TP in this study.

The fourth limitation is a relatively high rate of TP automatic segmentation failure (~24%; 10 of 41 subjects). The rate of labeling error by FreeSurfer increases as the size of an ROI decreases (Desikan et al., 2006). Future studies using higher resolution images with less partial volume effect might be needed to avoid parcellation error.

The fifth limitation concerns our inclusion of a limited number of subcortical structures—the amygdala and hippocampus—as target regions. Other subcortical regions, such as the nucleus accumbens, deserve attention due to their robust connection with the temporal pole in monkeys, as revealed by tract-tracing (Choi et al., 2017). Such connections merit further investigation in future *in vivo* human studies.

Other limitations include clear difference in sex ratio in the subjects (28 males and 3 females), relatively low imaging quality of our data compared with such data as the Human Connectome Project, and the absence of comparisons to monkey fiber tracing experiments.

Finally, we only investigated the left TP, which was chosen because of evidence for its prominent involvement in semantic dementia (Binney et al., 2010; Olson et al., 2013). However, to fully understand the roles that the TP plays in semantic processing, investigation of the bilateral TP is necessary and should be performed in future studies.

## Conclusion

Using diffusion MRI, we revealed that the TP has more extensive structural connectivity than has been reported previously, and that each of the cytoarchitectonically distinct TP subregions have partially overlapping yet distinct patterns of structural connectivity with areas outside of the TP. We propose that the extensive structural connectivity revealed in this study is suggestive of the TP playing the role of a brain convergence zone where the information integration occurs. Our findings may help in understanding the progression of diseases such as semantic dementia, which originates in the TP with the pathogens likely spreading through anatomical connections to other brain areas. The structural connectivity of the TP needs to be explored further in future studies using higher resolution, higher signal-to-noise acquisition techniques.

## Data availability statement

The datasets presented in this article are not readily available because participants did not provide consent to open data sharing. However, the datasets generated during the current study are available from the corresponding author on reasonable request. Requests to access the datasets should be directed to MK, kubicki@bwh.harvard.edu.

## Ethics statement

The study involving humans was approved by the local IRB committees at Brigham and Women’s Hospital and VA Boston. The study was conducted in accordance with the local legislation and institutional requirements. The participants provided their written informed consent to participate in this study.

## Author contributions

TS, NM, BD, and MK designed the experiment. SB collected data and assisted imaging preprocessing analyses. TS performed data analyses with feedback from NM, PS, YR, RE, MS, BD, and MK. TS, MK, and EY wrote and revised the manuscript. All authors contributed to the article and approved the submitted version.

## Footnotes

^1^ http://surfer.nmr.mgh.harvard.edu

^2^ http://www.slicer.org

^3^ http://www.fmrib.ox.ac.uk/fsl

^4^ https://github.com/pnlbwh/ukftractography

^5^ http://www.r-project.org
